# Anthocyanin Profiles in Colored Potato Tubers at Different Altitudes by HPLC–MS Analysis with Optimized Ultrasound-Assisted Extraction

**DOI:** 10.3390/foods12224175

**Published:** 2023-11-20

**Authors:** Zheying Qi, Weilu Wang, Zhen Liu, Na Niu, Zhitao Li, Limin Chen, Jinyong Zhu, Dechen Li, Yuhui Liu

**Affiliations:** 1 Agronomy College, Gansu Agricultural University, Lanzhou 730070, China; qzygsau@foxmail.com (Z.Q.);; 2 State Key Laboratory of Aridland Crop Science, Gansu Agricultural University, Lanzhou 730070, China

**Keywords:** potato, anthocyanin, ultrasound-assisted extraction, response surface methodology, HPLC–MS

## Abstract

The elevated anthocyanin content of colored potatoes produces numerous health benefits in humans. However, there is a paucity of studies exploring the influence of environmental factors on anthocyanin components in colored potatoes. In our work, the Box–Behnken design was adopted to optimize anthocyanin extraction from colored potato tubers with ultrasound assistance. The response surface model was stable and reliable (R^2^ = 0.9775), and under optimal extraction conditions, namely an ultrasonic power of 299 W, an extraction time of 10 min, and a solid-liquid ratio of 1:30 (g/mL), the yield reached 4.33 mg/g. Furthermore, the anthocyanins of colored potato tubers grown at different altitudes were determined by high-performance liquid chromatography–mass spectrometry with optimized ultrasound-assisted extraction, the results showed that anthocyanin levels were the highest at high altitudes, whereas anthocyanins were almost undetectable at mid-altitude. Moreover, the types of anthocyanin compounds present in colored potatoes varied at different altitudes. The red clones exhibited substantial accumulation of pelargonidin across all three altitudes. In contrast, the main anthocyanins found in purple clones were malvidin, petunidin, and cyanidin. We identified the anthocyanin components with a strong correlation to the environment, thereby establishing a fundamental basis for the breeding of potato clones with high anthocyanin content.

## 1. Introduction

Potato (*Solanum tuberosum* L.) is an annual crop in the family Solanaceae. The tuber has wide adaptability and is grown in more than 150 countries as it is rich in many nutrients and has a high and stable yield [1]. Most potato cultivars have white or yellow flesh, some called colored potatoes have red, purple, blue, and black flesh. Colored potatoes contain all of the nutrients of white or yellow potatoes, such as starch, edible fiber, protein, amino acids, and vitamin C. In addition, colored potatoes are rich in antioxidants and anthocyanin glycosides [2]. Anthocyanins are the main subclass of colored flavonoids and consist of red, purple, or blue pigments found in various plants [3]. They exhibit several physiological functions, such as scavenging free radicals and antioxidants [4], enhancing immunity, and slowing down aging [5,6]. In addition, anthocyanins can enhance the ability of plants to resist adversity [7]. They are expected to replace synthetic pigments in the food and medical fields with promising application potential [8].

Currently, the traditional solvent extraction method is most often used to extract anthocyanins; however, because of its long duration and low efficiency, this method may lead to anthocyanin degradation as prolonged thermal exposure decreases the extract’s antioxidant activity [8,9]. Ultrasound-assisted extraction (UAE) uses the effects of vibration and cavitation produced by ultrasound to increase the speed of the extraction solvent into cells [10] so that the required active ingredients can be fully extracted [11]. Compared with conventional solvent extraction, UAE is rapid, more efficient, solvent-saving, and time-saving. Therefore, UAE was widely used for extracting natural compounds from plants [12,13].

Response surface methodology (RSM) determines the optimal conditions for a multivariable system by evaluating the relationship between factors and response values that have been widely applied in the process industry to optimize various parameters [14]. RSM can determine the optimal extraction conditions and determine whether the selected independent variables significantly affect the response [15]. Many methods have been established to detect anthocyanins, such as nuclear magnetic resonance (NMR) spectrometry [16], high-performance liquid chromatography (HPLC) [17], and high-performance liquid chromatography–mass spectrometry (HPLC–MS) [18]. Among these methods, HPLC–MS is used in the analysis and detection of anthocyanins due to its advantages of high selectivity, high sensitivity, and quantitative accuracy [19,20]. However, there is a scarcity of studies regarding anthocyanin analysis in potato extracts because of the inherent instability and susceptibility of anthocyanins to environmental influences [21]. Most studies on colored potato tuber extracts have focused on compositional analysis and antioxidants, but studies on environmental factors affecting anthocyanin content and components of colored potato are few.

This study aimed to optimize UAE process parameters, including ultrasonic power, extraction time, and solid-liquid ratio, and use RSM to determine the best extraction method for anthocyanins from colored potato tubers. Furthermore, anthocyanins in the tuber extracts of six potato clones grown at high, middle, and low altitudes were analyzed by the optimized extraction method and HPLC–MS to investigate the influence of altitudes on potato tuber anthocyanin content and components. According to the observed variations in anthocyanin composition among different potato clones grown at different altitudes, we stratified and clustered quantitative data on 28 anthocyanins, and identified anthocyanin components that were significantly affected by environmental factors. This work will establish a foundation for future investigations into the molecular regulatory mechanisms of anthocyanin biosynthesis in potato tubers associated with environmental changes.

## 2. Materials and Methods

### 2.1. Plant Materials

The potato material used for optimizing anthocyanin extraction by RSM was purple clone CIP302285.27 (annotated as P). Three purple clones, CIP302288.14, CIP302288.35, and CIP302288.39; one yellow clone, CIP302286.26; and two red clones, CIP302284.17 and CIP302286.13 were used for assessing anthocyanin content and conducting HPLC–MS component analysis. These potato clones were obtained from Hebei North University (Zhangjiakou City, Hebei Province, China).

### 2.2. Sample Preparations

A mixed sample of five tubers per plant was taken at harvest for a total of three plants. Potatoes were peeled with a scalpel and cut into thin slices of about 3 mm. The samples were lyophilized over 48 h (Alpha 1–2 LD plus; Martin Christ GmbH, Osterode am Harz, Germany), then crushed and mixed in a mixer mill (MM400; Retsch GmbH, Haan, Germany). Completely crushed samples were collected and frozen at −80 °C until use.

### 2.3. Data on Climatic Factors

P was planted in Dingxi City in Gansu Province, while the other potato clones were grown at three locations: low altitude in Libo County (Qiannan Autonomous Prefecture, Guizhou Province, altitude 800 m); middle altitude in Dingxi City (Gansu Province, altitude 1600 m); and high altitude in Lasa City (Tibet Autonomous Region, altitude 3600 m). Comprehensive climatic data of the three sites are available in Liu’s work [22].

### 2.4. Ultrasound-Assisted Extraction

Ultrasound-assisted extraction of anthocyanins was carried out based on previously reported literature with some modifications [23]. The ultrasound-assisted extraction (UAE) was carried out in an ultrasonic device (SCIENTZ-IID, Ningbo Scientz Biotechnology Co., Ltd, China) with an ultrasound power of 1000 W and an ultrasound probe tip with a shaft diameter of 6 mm. The frequency was 20–25 kHz frequency automatic tracking, and a Φ6 variable amplitude pole was used for extraction. Subsequently, purple potato powder was accurately weighed in a centrifuge tube to 0.1 g and added to the extraction solution (0.1% HCl and 95% ethanol, volume 1:1). The sample was subjected to centrifugation at 4000 rpm for a duration of 10 min to obtain a supernatant containing anthocyanin extracts.

### 2.5. Total Anthocyanin Quantification

The total anthocyanin of purple potato was performed using a UV spectrophtometer at different wavelengths as described by Nunes Mattos and Ticconi [24,25]. A potato anthocyanin extract of 1 mL was placed in a test tube. It was diluted to 10 mL with two kinds of pH buffers (pH 1.0 and 4.5), respectively. The absorbance readings of both buffers were recorded at wavelengths of 530 and 700 nm, respectively. Each sample was measured three times concurrently, the average value was taken to calculate the total anthocyanin content (TAC) of the potato according to the following equation:

$$\mathrm{TAC}\;(\mathrm{mg}/g)=\frac{\left[\left(A_{530}\;-\;A_{700}\right)\mathrm{pH}\;1.0\;-\;\left(A_{530}\;-\;A_{700}\right)\mathrm{pH}\;4.5\right]\;\times \;M\;\times \;\mathrm{DF}\;\times \;V}{\varepsilon \;\times \;L\;\times \;m}$$

In the formula provided, A_530_ and A_700_ represent the absorbencies at the wavelengths of 530 nm and 700 nm, respectively. V represents the volume of the diluted solution measured in milliliters, while M denotes the molecular weight of cyanidin-3-glucoside, which is 449.2 g/mol. The dilution factor is represented by DF, and ε represents the molar extinction coefficient of cyanidin-3-*O*-glucoside, which is 26900 L/mol/cm. Additionally, the optical diameter of the cuvette is denoted by L in centimeters, and the sample weight is represented by m in grams.

### 2.6. Single-Factor Tests for Total Anthocyanin Extraction

The ionic nature of anthocyanins makes them sensitive to changes in pH, and they are usually extracted with acidic solvents under mild conditions [26]. Therefore, this study used 0.1% HCl and 95% ethanol, volume 1:1 as the initial extraction solvent. The effect of each single factor condition on anthocyanin content was studied under different experimental conditions. Based on the highest value of total anthocyanin content (expressed as mg cyanidin-3-glucoside by weight of purple potato), the optimum conditions of three single factors: extraction power, extraction time, and solid-liquid ratio, were optimized to obtain the highest anthocyanin content of potato tuber. Specific conditions for the single-factor tests were as follows: the ultrasonic powers were set to 150, 200, 250, 300, and 350 W, the extraction times were set to 5, 10, 15, 20, and 25 min, and the solid-liquid ratio is an important parameter to reduce cost and increase extraction rate. Therefore, we mixed 1 g of sample with the extraction solvent in the ratio of 1:10, 1:15, 1:20, 1:25, and 1:30 g/mL for the solid-liquid ratio investigation, respectively. Each single-factor experiment contained three replicates to determine the best value.

### 2.7. Optimization of Anthocyanin Extraction

The response variables were usually influenced by an interaction of independent variables, so the relationship between three single factors (extraction power, ultrasonic time, and solid-liquid ratio) and anthocyanin yield (*Y*) was designed using Box–Behnken to optimize and determine the optimum value. A total of 17 randomized runs were conducted in the experiment. Following is a list of the independent variables and their levels (Table 1): A is the ultrasonic power (W): 300, 250, 200; B is the extraction time (min): 20, 15, 10; and C is the solid-liquid ratio (g/mL): 1:30, 1:25, 1:20. The three experimental factors were coded by placing them at three levels (1, 0, −1). To reduce the impact of system errors on the observed reactions, the experimental run was completely randomized. The following equation was used to code variables:

$$x=(X_{i}-X_{0})/\Delta X$$

where x is the encoding value, $X_{i}$ is the corresponding actual value, $X_{0}$ is the actual value of the domain center, and ΔX represents the increment of the independent variable x. The second-order polynomial model developed based on Box–Behnken is here,

(1) $$Y=\beta_{0}+\sum_{i=1}^{3}\beta_{i}X_{i}+\sum_{i=1}^{3}\beta_{ii}X_{i}^{2}+\sum_{i=1}^{2}\sum_{j=i+1}^{3}\beta_{ij}X_{i}X_{j}$$

*Y* is the response variable to be modeled (anthocyanin yield), $\beta_{0}$ is the constant coefficient, $\beta_{i}\;$is the linear coefficient, $\beta_{ii}$ is the coefficient of quadratic effect, $\beta_{ij}$ is the interactive effects of the variables, and $X_{i}\;{and\;X}_{j}$ are the independent variables.

### 2.8. Purification of Anthocyanins from Potato Tubers

The anthocyanin extract was vacuum-concentrated by evaporation at 40 °C under a rotary evaporator (Büchi, New Castle, DE, USA). It was reconstituted with 5 mL of chromatographic grade methanol solution, then filtered using a 0.22 µm microporous filter, and finally stored in a brown injection flask, which was stored at 4 °C for component analysis.

### 2.9. Identification of Anthocyanins Using HPLC–MS

HPLC–MS was used for the determination and analysis of anthocyanin components in colored potato extracts. Data were acquired using a liquid chromatography–triple quadrupole mass spectrometry system (Agilent Technologies, Palo Alto, CA, USA). The samples were separated on an Acquity HPLC HSS T3 (C18) column (2.1 × 100 mm, 1.8 μm; Waters Corporation, Milford, MA, USA) using ultrapure water and acetonitrile solution both contained 0.04% acetic acid as eluent A and eluent B. The program of elution was used as follows: 5% B at the time of injection; B changed linearly from 5% to 95% at 1 min, maintained at 95% B for 1 min. B decreased to 5% from 11.00 to 11.10 min and remained balanced at the 5% level until 14.00 min. The flow rate and the column temperature were maintained at 0.35 mL/min and 40 °C, respectively; the injection volume was 10 μL.

### 2.10. Statistical Analysis

Each experiment was performed three times and experimental data were presented as the mean ± standard deviation (mean ± SD) deviation. One-way analysis of variance (ANOVA) and multiple comparison tests were performed using SPSS software (v26.0; SPSS Inc., Chicago, IL, USA). *p* < 0.05 was considered significant, whereas *p* < 0.01 was considered highly significant. Heat map of anthocyanin peak areas at different altitudes using TBtools software (https://github.com/CJ-Chen/TBtools (accessed on 20 June 2023)).

## 3. Results

### 3.1. Single-Factor Analysis of Anthocyanin Extraction

**Figure 1:**
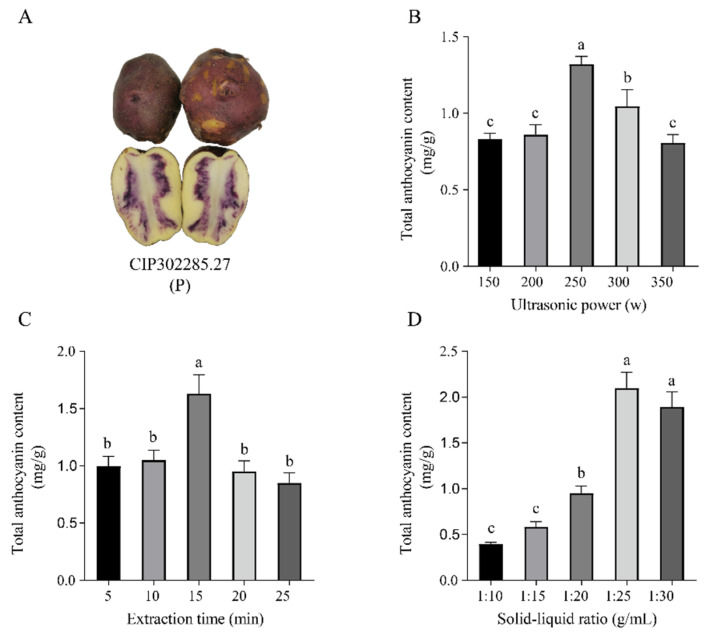
Single-factor tests for anthocyanin extraction in colored potato clone P. (**A**) Potato tuber of clone CIP302285.27 (annotated as P) grown in Dingxi (1800 m). (**B**) Effects of ultrasonic power in the range of 150–350 W on total anthocyanin content (TAC), (**C**) Effects of extraction time from 5 to 25 min on TAC, (**D**) effects of solid-liquid ratio in a range of 1:10–1:30 (g/mL) on TAC. Error bars indicate the standard deviations of the samples. Different Superscript letters (a, b, c) indicate statistically significant differences among groups (*p* ≤ 0.05).

The purple potato clone P grown in Dingxi City was used for single-factor tests to assess various factors that affect the anthocyanin extraction yield of tuber flesh (Figure 1A). The results showed that in the first single-factor test, the total anthocyanin content increased gradually as the ultrasonic power increased from 150 to 250 W. The maximum anthocyanin yield of potato tubers was obtained when the ultrasonic power reached 250 W. However, ultrasonic power higher than 250 W resulted in anthocyanin degradation in potatoes (Figure 1B). The second single-factor test result showed that the total anthocyanin content increased to a maximum of 1.63 mg/g at 15 min, followed by a gradual decrease (Figure 1C), indicating the optimum extraction time for anthocyanins in potato tuber was 15 min. Following these observations, further experiments were performed with 250 W ultrasonic power and 15 min extraction time. In the third single-factor test, the total anthocyanin content gradually increased when the solid-liquid ratio ranged from 1:10 g/mL to 1:25 g/mL, and decreased as the solid-liquid ratio increased from 1:25 g/mL to 1:30 g/mL, the highest anthocyanin content reached to 2.04 mg/g when the solid-liquid ratio was 1:25 g/mL (Figure 1D). Thus, the optimal parameters for anthocyanin extraction in potato tuber were as follows: 250 W ultrasonic power, 15 min extraction time, and a solid-liquid ratio of 1:25 g/mL.

### 3.2. Regression Equation Establishment and Analysis of Variance

According to the single-factor tests, three independent variables: ultrasonic power (A), extraction time (B), and solid-liquid ratio (C) were used to optimize the response surface, the response value was the anthocyanin content (Y) of potato tuber. The experimental design and results are summarized in Table 2. Experimental data were fitted with quadratic multiple regression using Design-Expert 13.0 software, and the regression equation between the independent variables and the response values was obtained as follows:

$$Y=3.26\;-\;0.0912A+0.1450B+0.9213C+0.0625\mathrm{AB}+0.4000\mathrm{AC}\;-\;0.4075\mathrm{BC}+0.1120A^{2}\;-\;0.0505B^{2}\;-\;0.4430C^{2}$$

The ANOVA of the above regression equation is shown in Table 3. The model’s F-value of 33.83 and the respective *p*-value of 0.0001 signified the model’s high level of significance. The model determination coefficient (R^2^) of 0.9775 and the adjusted determination coefficients (Adj R^2^) of 0.9486 indicated a good correlation and good fitting effect of the model. The lack-of-fit F-value of 2.67 and *p*-value of 0.1836 indicated the small error between the experimental results and the predicted values, further confirming that the model was well fitted. Thus, the model can be used to calculate the experimental results.

The partial regression coefficient of C was highly significant (*p* < 0.0001), indicating the solid-liquid ratio significantly influenced anthocyanin extraction from potato tuber. In contrast, the coefficients of A and B were not significant, indicating the ultrasonic power and extraction time had minimal influence on anthocyanin extraction from potato tuber. (*p* > 0.05). The interactive terms AC and BC and the quadratic term C^2^ also showed a significant impact on anthocyanin extraction with *p* < 0.05.

**Figure 2:**
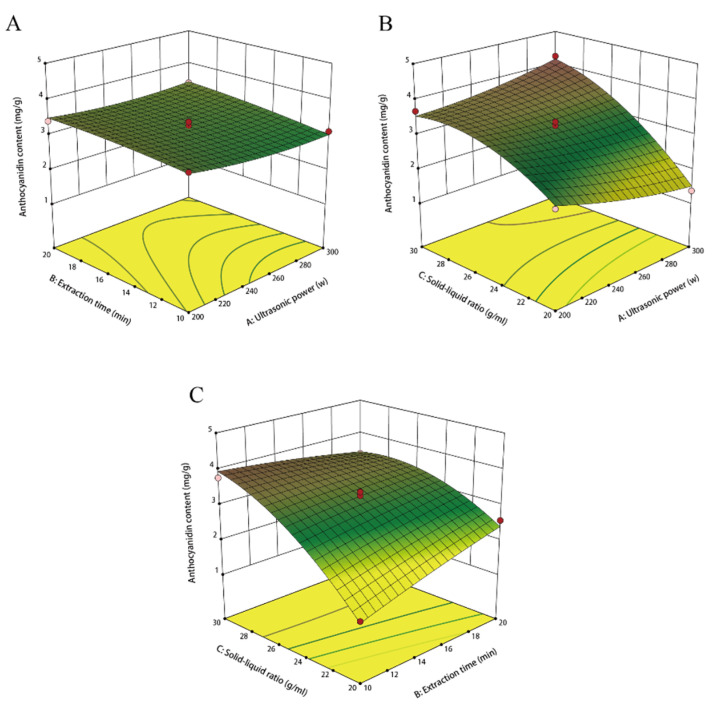
Response surface of anthocyanin content affected by the interactions among three independent variables. (**A**) Interactions between ultrasonic power and extraction time. (**B**) Interactions between ultrasonic power and solid-liquid ratio. (**C**) Interactions between extraction time and solid-liquid ratio.

It is particularly important to understand the interaction of extraction factors with response value. In our work, response surface plots were constructed to illustrate the effects of the interaction of two factors on the anthocyanin content, with one factor fixed at a constant central value. The results showed the impact of individual factors on anthocyanin yields. In this way, it was found that there are insignificant influences of the interaction of ultrasonic power and extraction time on anthocyanin contents in colored tuber flesh at a solid-liquid ratio of 1:25 g/mL (Figure 2A). Whereas the interaction effect of ultrasonic power and solid-liquid ratio on anthocyanin yield was significant when the extraction time was maintained at 30 min (Figure 2B). At the same ultrasonic power of 300 W, with the increase in the solid-liquid ratio, the anthocyanin yield of colored potato gradually increased, and the anthocyanins reached the maximum of 4.33 mg/g when the solid-liquid ratio was 1:30 g/mL (Figure 2C). It was seen that the interaction between the two factors, ultrasound time and solid-liquid ratio, was strong and had a more significant effect on the extraction of potato anthocyanin.

### 3.3. Model Optimization and Verification

The optimal extraction conditions for colored potato anthocyanins with the highest extraction rate were predicted using Design-Expert software based on response surface experimental data: ultrasonic power was 299.9 W; extraction time was 10 min; and solid-liquid ratio was 1:30 (g/mL). The predicted maximum anthocyanin yield was 4.304 mg/g. To verify whether the model is feasible, we performed three parallel verification experiments under optimum conditions consisting of an ultrasonic power of 300 W, an extraction time of 10 min, and a solid-liquid ratio of 1:30 g/mL. The average anthocyanin yield was 4.33 mg/g. Thus, the results can be reliably utilized for optimized anthocyanin extraction from colored potato tuber flesh.

### 3.4. Anthocyanin Yield Obtained Using the Optimized Ultrasonic Method

**Figure 3:**
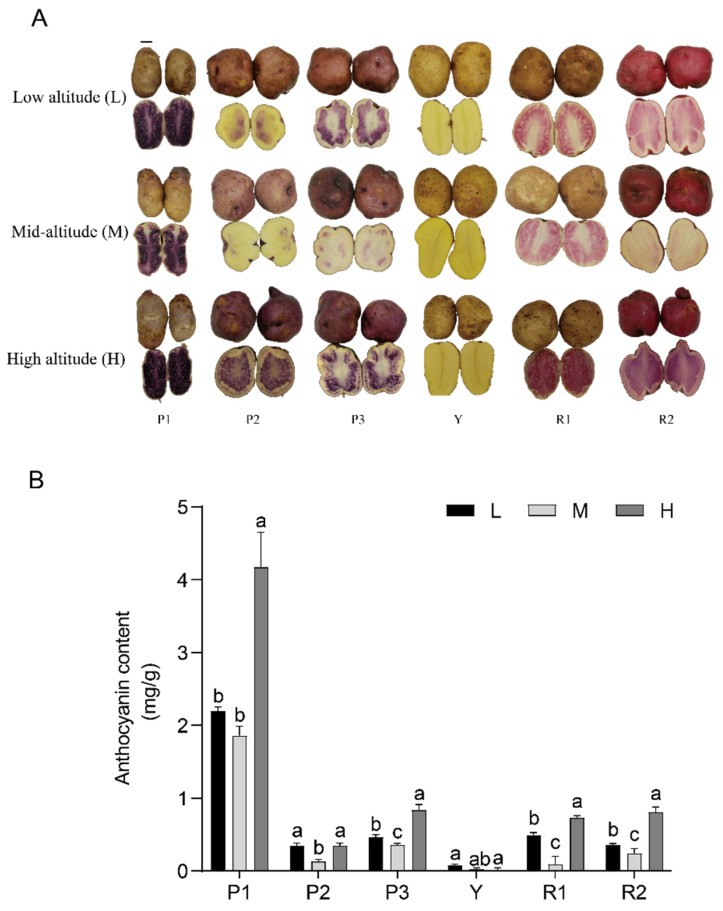
Anthocyanin content and tuber color of six potato clones grown at high altitude, mid-altitude, and low altitude. (**A**) Changes in color among potato tubers from clones at different altitudes. Purple clones CIP302288.14, CIP302288.35, and CIP302288.39 were annotated as P1, P2, and P3, yellow clone CIP302286.26 was renamed Y, and red clones CIP302284.17 and CIP302286.13 were annotated as R1 and R2. Scale bar = 2 cm. (**B**) Differences in anthocyanin components among clones at different altitudes. L: low altitude (800 m), M: mid-altitude (1800 m), and H: high altitude (3600 m). Error bars indicate the standard deviations of the samples. Different Superscript letters (a, b, c) indicate statistically significant differences among groups (*p* ≤ 0.05).

To validate the optimization method, we selected six potato clones grown at different altitudes (Figure 3A). In colored tubers, anthocyanin content was highest at high altitudes and lowest at mid-altitude among all colored potatoes. In contrast, the yellow-skinned, yellow-fleshed potatoes Y, anthocyanin was almost undetectable at three different altitudes. Variations in the anthocyanin content at various altitudes are shown in Figure 3B. The anthocyanin yield of the colored clones at high altitude was in descending order of magnitude P1 > P3 > R2 > R1 > P2 > Y, and their levels were 4.173, 0.835, 0.806, 0.730, and 0.349 mg/g, respectively. At mid-altitude, the anthocyanin yield of the colored clones was in the order P1 > P3 > R2 > P2 > R1 > Y, and their corresponding levels were 1.858, 0.358, 0.244, 0.132, and 0.091 mg/g, respectively. The anthocyanin yield of the colored clones at low altitude was in the order P1 > R1 > P3 > R2 > P2 > Y, and their levels were 2.197, 0.492, 0.465, 0.358, 0.345, and 0.076 mg/g, respectively. Among these, the purple potato P1 showed a significantly higher anthocyanin content than the other potatoes at all three altitudes (*p* < 0.05), whereas the anthocyanin content of other clones varied at different altitudes, and the anthocyanin accumulation extent is not consistent with the altitude changes.

### 3.5. Analysis of Anthocyanin Composition of Potatoes at Different Altitudes Using HPLC–MS

**Figure 4:**
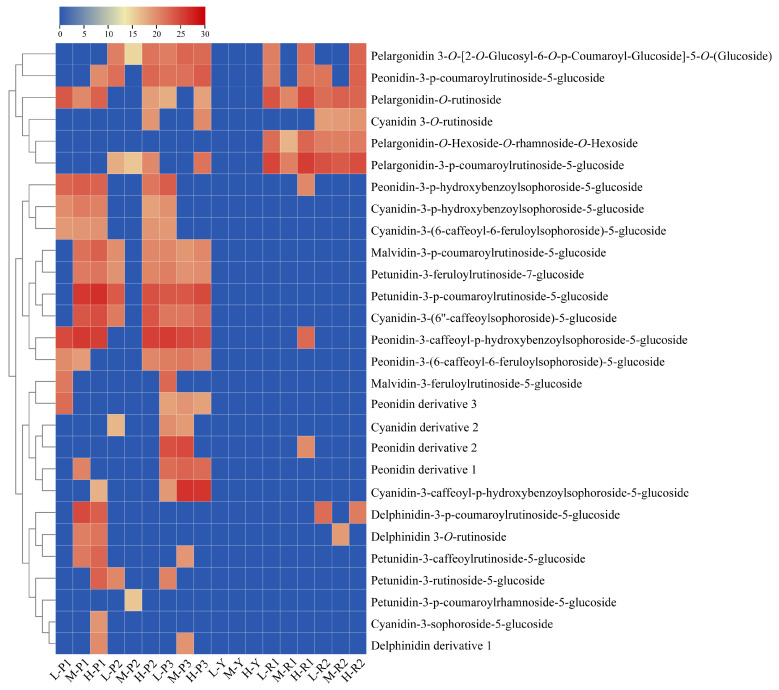
Differences in anthocyanin components and contents among clones at different altitudes. TBtools software was used to generate this figure, and the color key represents log2 transformed expression levels from high (red) to low (blue).

All extracts were analyzed by HPLC–MS to explore the effects of different altitudes on the anthocyanin composition of potatoes and to determine differences in their anthocyanin composition. The peak area and retention time of anthocyanin components of different clones at different altitudes are shown in Table S1. Table S2 shows the anthocyanin contents of colored potatoes at different altitudes as a percentage of total anthocyanins. A total of 30 compounds were detected in colored potatoes by HPLC–MS. Based on retention times as well as mass charge ratios, compared with the analysis of anthocyanin species in the existing literature, we deduced that 28 compounds. All anthocyanins detected were linked to a glycosyl group via a glycosidic bond to form an anthocyanin glycoside that glycosylated anthocyanins acylate to phenolic acids to form acylated anthocyanin glycosides. Among them, 23 anthocyanins have been reported in the literature, and 5 have not been characterized in the literature. We called them derivatives. Varietal discrepancies in anthocyanin components at various altitudes are illustrated in Figure 4. The effect of different altitudes on the anthocyanin components was investigated by comparing the peak areas. The red potato clones R1 and R2 at all three altitudes contained pelargonidin-*O*-rutinoside, pelargonidin-3-p-coumaroyl-rutinoside-5-glucoside, and pelargonidin-*O*-hexoside-*O*-rhamnoside-*O*-hexoside. At high altitudes, pelargonidin3-p-coumaroylrutinoside-5-glucoside in R1 and R2 accounted for the highest levels of total anthocyanins 50.65% and 43.88%, respectively. Therefore, we speculated that this component is the reason for the deepening of the color of the red clones. In contrast, the purple clones P1, P2, and P3 all contained malvidin-3-p-coumaroylrutinoside-5-glucoside, petunidin-3-feruloylrutinoside-7-glucoside, petunidin-3-p-coumaroylrutinoside-5-glucoside, and cyanidin-3-(6″caffeoyl-sophoroside)-5-glucoside, and the changes in these four components remain the same. In the same clone, with the change of altitude, anthocyanin components will also change. In the P1 and P2 clones, the content of petunidin-3-p-coumaroylrutinoside-5-glucoside under high, middle, and low altitude conditions was 0, 33.8%, 44.01%, and 49.55%, 0, 19.55%, respectively. The content of peonidin-3-caffeoyl-p-hydroxybenzoylsophoroside-5-glucoside was 47.32%; 31.39%; 19.87%; and 0, 0, 43.21%, respectively. From this, we could deduce that the purple of the P2 clone at high altitude was due to the high content of the component peonidin-3-caffeoyl-p-hydroxybenzoylsophoroside-5-glucoside. Furthermore, cyanidin-3-sophoroside-5-glucoside exclusively existed in the purple clone P1 and was not detected in other clones.

## 4. Discussion

Anthocyanins belong to a family of bioactive compounds called flavonoids that endow plants with diverse hues [27]. These bioactive compounds exhibit significant nutritional and health-promoting properties in the human body. They possess anti-aging properties, which help in the prevention of cardiovascular ailments while enhancing blood glucose and visual acuity [5]. Anthocyanin-rich plants can be developed into functional foods and therapeutic medicines.

In this study, UAE, a widely used extraction technique, was optimized using a combination of single-factor tests and RSM. In potato tubers, anthocyanins are mainly extracted by solvent extraction; however, the anthocyanin content obtained by UAE is obviously higher than the traditional solvent extraction method [28]. UAE offers notable advantages of higher extraction yields, shorter extraction time, and lower extraction temperature compared with the traditional maceration method [12]. The extraction efficiency in ultrasonic-assisted extraction is affected by multiple parameters, mainly by solid-liquid ratio, extraction time, ultrasonic power, and extraction temperature [29]. Ultrasonic power can also significantly influence anthocyanin yield. The anthocyanin content reached its peak when the ultrasonic power was 250 W in our study (Figure 1B). This phenomenon was because of the cavitation effects of ultrasound in the solvent and local pressure, resulting in the solvent penetrating deeper into potato tuber cells, accelerating the release of intracellular anthocyanins, promoting their entry into solvents, and fully dissolving, which increased anthocyanin production [30]. However, anthocyanin yield decreased when the ultrasonic power exceeded 250 W, maybe because of the high ultrasonic power overheating the reaction system, leading to anthocyanin degradation. This observation aligns with the trend in the ultrasonic power required to extract anthocyanins from araticum and mandarin [31,32]. Extraction yields increased when increasing the extraction time from 5 to 15 min, (Figure 1C). This was because of the low diffusion resistance of intracellular anthocyanins during the early stages of extraction caused by ultrasound damage to the cell structure, which facilitates anthocyanin extraction. However, when the time was increased to 25 min, the anthocyanin yield decreased, this may be because the increased extraction time caused the anthocyanin molecules to decompose, thereby reducing the light absorption value. When the extraction time exceeded 25 min, anthocyanin content showed a decreasing tendency, suggesting that excessive extraction time under sonication conditions might lead to oxidative anthocyanin degradation, which is in line with the previous report [33]. It is reported that the increase in solute–solvent interactions resulted from the increase in the concentration gradient [34]. In our study, the content of extracted anthocyanins increased as the solid-liquid ratio increased from 1:10 g/mL to 1:25 g/mL. However, the anthocyanin content decreased slightly as the solid-liquid ratio increased to 1:30 g/mL, which could indicate the saturation of the extraction medium. We utilized RSM to gain a more comprehensive understanding of how various parameters impact anthocyanin content. Anthocyanin extraction was optimized based on single-factor tests and three-level, three-factor RSM, which is a statistical approach used for the design and development of experiments, the building of models, the evaluation of the effects of processing parameters on responses, as well as the optimization of processes [35]. The degree of interaction between variables can be evaluated by examining the slope of the 3D response surface, a steeper slope indicates a more pronounced interaction between the variables. In addition, on a 2D surface, the color of the response surface provides an indication of the optimum conditions. The color tended to change from yellow to red with the increase in yield. The trends in the independent variables (power, time, and solid-liquid ratio) were distinct. Specifically, an increase in solid-liquid ratio resulted in a significantly enhanced anthocyanin yield. Hence, the optimal extraction parameters for anthocyanins from colored potatoes consist of an ultrasonic power of 300 W, an extraction time of 10 min, and a solid-liquid ratio of 1:30 g/mL. The anthocyanin content extracted was 4.33 mg/g.

Potato tubers usually exhibit high anthocyanin content, which can be easily obtained. To capitalize on the availability of colored potatoes, enhance their value, and avoid resource wastage, anthocyanin extraction from potato tubers can offer a unique approach to better exploit and develop potato resources. Besides genetic components, environmental factors can also influence anthocyanin biosynthesis [36]. Temperature may play a vital role in regulating anthocyanin biosynthesis in potato tubers. Substantial diurnal thermal fluctuations and ample light at elevated altitudes promote the activity of enzymes related to anthocyanin and enhance the accumulation of anthocyanin in plants [37]. For example, anthocyanin content was increased in apples, purple rice, and rabbiteye blueberry grown at high altitudes [38,39,40]. In our work, we used the optimized UAE method to ascertain the anthocyanin content of six clones grown at varying altitudes. The anthocyanin content was at its peak in all six clones at high altitudes, which is consistent with previous findings. Interestingly, the anthocyanin content was lower at mid-altitude than at low altitude, this may be because both high and low altitudes exhibit low air and solid temperatures during the tuber enlargement phase [22], and exactly which environmental factors influenced this requires further study.

To validate the results of the UAE method, we identified the anthocyanin components of six colored potato clones at different altitudes using HPLC–MS. In total, 28 anthocyanin compounds were detected, and pelargonidin derivatives were the main anthocyanin compounds in the two red clones. Anthocyanin derivatives, mainly malvidin, peonidin, cyanidin, and petunidin, were greater in purple clones than in red clones. Higher levels of cyanidin, delphinidin, and peonidin were found in red clones, and higher levels of malvidin and petunidin were found in purple clones. Petunidin and malvidin derivatives dominated the purple varieties, while pelargonidin derivatives dominated the red varieties [41]. In addition, purple clones contained mainly anthocyanin glycosides, namely malvidin-3-p-coumaroyl-rutinoside-5-glucoside [42]. Our study also reached the same conclusion. In red potatoes, pelargonidin-3-p-coumaroyl-rutinoside-5-glucoside was detected [43,44]. In this study, pelargonidin-3-p-coumaroyl-rutinoside-5-glucoside was the main derivative in the red clones. In our study, the contents of petunidin-3-p-coumaroylrutinoside-5-glucoside and peonidin-3-caffeoyl-p-hydroxybenzoylsophoroside-5-glucoside in the three high-altitude purple clones were higher than the other components, so we speculated that the darker color of the purple varieties at high altitudes was due to these two components. Similarly, we speculated that the deepening of the color of the red variety at high altitudes was due to pelargonidin-*O*-rutinoside and pelargonidin-3-p-coumaroylrutinoside-5-glucoside. Therefore, based on the HPLC–MS analysis results, our method of the UAE can be efficiently utilized for the extraction of anthocyanins and the detection of anthocyanins with more sensitivity in potatoes. The findings lend credence to the practical application of anthocyanin resources found in potatoes with unique pigmentation. This has significant implications for the development of clones with diverse color variations that are suitable for various geographical regions.

## 5. Conclusions

Anthocyanins in colored potatoes were extracted by the UAE method. The extraction conditions were optimized by the Box–Behnken experimental design. The anthocyanin extraction yield was further optimized by RSM on the basis of single-factor experiments. Meanwhile, a quadratic polynomial regression extraction model was developed, which shows a higher coefficient of determination for the optimum conditions. UAE was optimized by single-factor tests combined with RSM. The optimal parameters were an ultrasonic power of 299 W, an extraction time of 10 min, and a solid-liquid ratio of 30 (g/mL). The optimum experimental yield of anthocyanins (4.33 mg/g) agreed closely with the predicted yield (4.304 mg/g). HPLC–MS analysis results showed that the red clones exhibited substantial accumulation of pelargonidin across all three altitudes. In contrast, the main anthocyanins found in purple clones were malvidin, petunidin, and cyanidin. The findings lend credence to the practical application of anthocyanin resources found in potatoes with unique pigmentation. This has significant implications for the development of clones with diverse color variations that are suitable for various geographical regions.

## Figures and Tables

**Table 1:** Coded and actual levels of the three variables.

Factor Levels	Independent Variable		
	Ultrasonic Power (A)	Extraction Time (B)	Solid-Liquid Ratio(C)
1	300	20	1:30
0	250	15	1:25
−1	200	10	1:20

**Table 2:** Response surface design experiments and anthocyanin content.

Run	Extraction Condition			Anthocyanin Content (mg/g)	
	A	B	C	Actual Value	Predicted Value
1	1 (300)	−1 (10)	0 (1:25)	3.11	3.02
2	1 (300)	1 (20)	0 (1:25)	3.4	3.44
3	1 (300)	0 (15)	1 (1:30)	4.24	4.15
4	0 (250)	1 (20)	−1 (1:20)	2.57	2.39
5	−1 (200)	1 (20)	0 (1:25)	3.4	3.49
6	0 (250)	0 (15)	0 (1:25)	3.11	3.26
7	−1 (200)	0 (15)	−1 (1:20)	2.41	2.49
8	0 (250)	−1 (10)	−1 (1:20)	1.34	1.29
9	0 (250)	−1 (10)	1 (1:30)	3.77	3.94
10	1 (300)	0 (15)	−1 (1:20)	1.37	1.51
11	−1 (200)	−1 (10)	0 (1:25)	3.36	3.33
12	0 (250)	1 (20)	1 (1:30)	3.37	3.42
13	0 (250)	0 (15)	0 (1:25)	3.38	3.26
14	0 (250)	0 (15)	0 (1:25)	3.39	3.26
15	0 (250)	0 (15)	0 (1:25)	3.13	3.26
16	0 (250)	0 (15)	0 (1:25)	3.27	3.26
17	−1 (200)	0 (15)	1 (1:30)	3.68	3.54

**Table 3:** ANOVA results of the response surface quadratic model.

Source	Sum of Squares	DF	Mean Square	F-Value	*p*-Value	Remarks
Model	9.22	9	1.02	33.83	<0.0001	significant
A	0.0666	1	0.0666	2.20	0.1817	
B	0.1682	1	0.1682	5.55	0.0506	
C	6.79	1	6.79	224.08	<0.0001	significant
AB	0.0156	1	0.0156	0.5157	0.4959	
AC	0.6400	1	0.6400	21.12	0.0025	
BC	0.6642	1	0.6642	21.92	0.0023	
A^2^	0.0528	1	0.0528	1.74	0.2283	
B^2^	0.0107	1	0.0107	0.3544	0.5704	
C^2^	0.8263	1	0.8263	27.27	0.0012	
Residual	0.2121	7	0.0303			
Lack of Fit	0.1414	3	0.0471	2.67	0.1836	not significant
Pure Error	0.0707	4	0.0177			
R^2^	0.9775					
R^2^ (Adj)	0.9486					
C.V.%	5.66					
Cor Total	9.44	16				

## Data Availability

The data presented in this study are available upon request from the corresponding author.

## Supplementary Materials

The following supporting information can be downloaded at: https://www.mdpi.com/article/10.3390/foods12224175/s1, Table S1: Anthocyanin mass spectrometry data of colored potatoes at different altitudes. Table S2: Anthocyanin contents of colored potatoes at different altitudes as a percentage of total anthocyanins.
